# Parametrizing analog multi-compartment neurons with genetic algorithms

**DOI:** 10.12688/openreseurope.15775.1

**Published:** 2023-09-08

**Authors:** Raphael Stock, Jakob Kaiser, Eric Müller, Johannes Schemmel, Sebastian Schmitt

**Affiliations:** 1 Kirchhoff Institute for Physics, Heidelberg University, Heidelberg, 69120, Germany; 2 European Institute for Neuromorphic Computing, Heidelberg University, Heidelberg, 69120, Germany; 3 Department for Neuro- and Sensory Physiology, University Medical Center Göttingen, Göttingen, 37077, Germany

**Keywords:** analog computing, neuromorphic, genetic algorithm, multi-compartment

## Abstract

**Background:** Finding appropriate model parameters for multi-compartmental neuron models can be challenging. Parameters such as the leak and axial conductance are not always directly derivable from neuron observations but are crucial for replicating desired observations. The objective of this study is to replicate the attenuation behavior of an excitatory postsynaptic potential (EPSP) traveling along a linear chain of compartments on the analog BrainScaleS-2 neuromorphic hardware platform.

**Methods:** In the present publication we use genetic algorithms to find suitable model parameters. They promise parameterization without domain knowledge of the neuromorphic substrate or underlying neuron model. To validate the results of the genetic algorithms, a comprehensive grid search was conducted. Trial-to-trial variations in the analog system are counteracted utilizing spike-triggered averaging.

**Results and conclusions:** The algorithm successfully finds solutions which replicate the desired EPSP attenuation behavior in both single and multi-objective searches illustrating the applicability of genetic algorithms to parameterize analog neuromorphic hardware.

## Introduction

Neuromorphic hardware describes beyond-von-Neumann computational systems, which try to implement computations in a way similar to the human brain. Often, these systems are capable of simulating neuron models, where the neurons communicate with each other in an event-based fashion using spikes. This makes neuromorphic hardware consume less energy compared to other systems ^1^. Many neuromorphic systems are also inherently scalable to larger systems ^2^. Whereby, neuromorphic computers differ in their physical realization ranging from purely digital ^3, 4^ over partially analog ^5^ up to complete analog systems ^6^. Digital realizations like SpiNNaker or Loihi focus on simulating spiking neural networks (SNNs) efficiently, for example by exploiting a network-on-a-chip architecture or by processing neural dynamics in an event-driven fashion. On the other hand, mixed-signal systems like BrainScaleS-2 (BSS-2) physically implement the neuron model, resulting in a continuous operation of the neuron in time.

In the case of BSS-2, neural time constants are usually a factor of 1000 smaller in comparison to their biological archetypes, resulting in a significant speed gain.

The hardware components which govern a neuron’s dynamics are configurable. Various conductances can be altered using bias currents in order to reach a desired operation point. Identically designed circuits deviate from each other in their real-world implementation because of mismatch during fabrication. This mismatch can be compensated by configuring the bias currents appropriately. Multiple chip components must be tuned to replicate high-level observations from biological neurons. However, these chip components can all influence observations in a highly nonlinear fashion.

In this paper, we demonstrate how genetic algorithms can be utilized to parameterize multi-compartmental neuron models on analog neuromorphic hardware. As an emulation platform BSS-2 will be used. The goal is to map high-level observations motivated by measurements of real neurons to the hardware. As methodology, we chose genetic algorithms since they outperformed conjugate-gradient descent and stochastic search as demonstrated by Vanier and Bower ^7^ in a task to constrain a multi-compartmental neuron model. Druckmann *et al.* ^8^ also successfully used genetic algorithms to parameterize multi-compartmental neuron models in simulation.

Previously, genetic algorithms proved their applicability in conjunction with various other neuromorphic hardware systems on many different problems. Elias and Chang ^9^ used a custom genetic algorithm on artificial dendritic trees, which are non-linear analog circuits, to determine the connectivity between neurons and sensory devices and the connectivity of the neurons themselves in order to classify a set of handwritten digits. Vandesompele *et al.* ^10^ used a genetic algorithm to find a network topology of an SNN on SpiNNaker such that the SNN could solve the exclusive or (XOR) problem. Furthermore, the authors used this algorithm to generate a functional SNN capable of playing the arcade game Pac-Man. Dalgaty *et al.* ^11^ proposed a concept, which they also verified in simulation, of a neuromorphic system for motion detection, where the underlying SNN was parameterized using genetic algorithms.

While computational neuroscience often focuses on point neuron models, in this paper we will investigate multi-compartmental neuron models. Real neurons in nature possess complex structure, which point neurons disregard. However, this structure is thought to play an essential role for the signal processing ^12^.

For example, the signal-receiving part of a nerve cell, called the dendritic tree, is thought to play an essential role in the signal-processing capabilities of a neuron ^13– 16^. Moreover, benefits when using dendrites are not limited to the level of single neurons but also the whole system can profit since dendrites could help to solve the credit assignment problem in biological brains ^17, 18^.

Morphological characteristics of neurons like dendrites can be realized on BSS-2 using a multi-compartmental neuron model. Multiple properties of the multi-compartment emulation on BSS-2, like passive attenuation properties but also active characteristics like back-propagating action potential activated calcium spike firing (BAC), were demonstrated in 19.

The following subsection will briefly introduce the hardware platform alongside the used multi-compartmental neuron model. In the “experiment” section, we present the task which the genetic algorithm will be applied to. After that, in the “methods” section, the genetic algorithms will be outlined. Subsequently, the results of the experiments are presented in the “results and discussion” section. Finally, in the “conclusions” section, the paper is concluded.

### BrainScaleS-2

**Figure 1.**
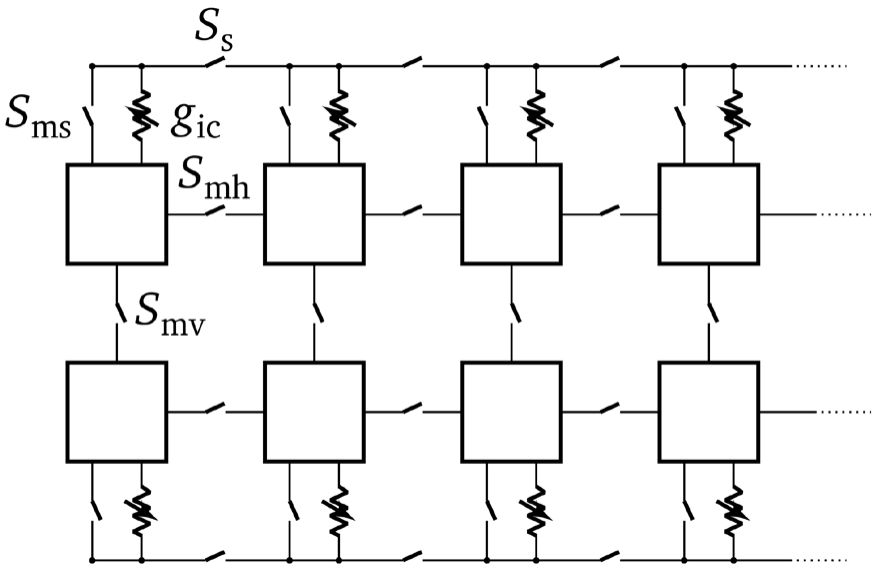
Overview of the connections available for implementing multi-compartmental neuron models on BrainScaleS-2. The squares illustrate neuron circuits which are physical implementations of the adaptive exponential integrate-and-fire neuron model. For more details refer to 5, 19.

BrainScaleS-2 is a mixed-signal neuromorphic chip that implements analog neurons and synapses. In total, there are 512 neurons implementing the adaptive exponential integrate-and-fire (AdEx) neuron model ^20, 21^. Those neurons are arranged in two rows with 256 neurons each. Above the upper neuron row there is a synapse matrix which can establish up to 256 synaptic connections per neuron circuit within that row. Similarly, there is a synapse matrix below the bottom neuron row. The AdEx model can be reduced to the simpler leaky integrate-and-fire (LIF) neuron model by simply disabling parts of the analog circuit ^20^. Through direct switches and adjustable conductances, single neuron circuits can be interconnected to form larger structured neurons (cf. Figure 1), resulting in a multi-compartmental model. Two possible types of connections exist. First, direct switches, which can be used to shorten a neuron’s membrane to its horizontal ( *S*_mh_) or vertical ( *S*_mv_) neighbors. Those switches can be used to increase the synaptic fan-in of a neuron. The second type of connection between neurons uses a shared line. Each neuron circuit can connect either *via* a switch ( *S*_ms_) or an adjustable conductance ( *g*_ic_) to that shared line. Furthermore, this shared line can be interrupted between neuron circuits using switches ( *S*_s_). A neuron can be separated into different compartments, by using the conductances *g*_ic_.

Besides the adjustable inter-compartment conductance, other properties of a neuron can be adjusted using various bias currents. The membrane voltage of a neuron can be recorded using the fast on-chip analog-to-digital converter (ADC) with a sampling frequency of 29MHz and a resolution of 10 bit ^22^. For a more in-depth description of the neuron circuit, refer to 5 or to 19 for more information about the multi-compartment features.

Experiments are formulated in the high-level neural network description language PyNN ^23^, which is supported by the software stack of BSS-2 ^24^.

### Multi-compartmental neuron model

The cable equation can be used to model passive charge transport in structured neurons. Segments of a neuron are represented as cylinders of fixed radii with constant electrical properties. Multiple cylinders can be concatenated to replicate a desired morphology ^25^. The one-dimensional cable equation is given by:


$$\tau_{\text{m}}\frac{\partial U_{\text{m}}(x,\;t)}{\partial t}-\lambda_{\text{m}}^{2}\frac{\partial^{2}U_{\text{m}}(x,\;t)}{\partial x^{2}}+U_{\text{m}}(x,\;t)=\frac{I^{\text{ext}}}{g_{1}}.\;(1)$$

Here *U*_m_( *x*, *t*) describes the membrane potential at position *x* at the time *t*, *τ*_m_ is the membrane time constant, *λ*_m_ is the electronic length scale, *g*_l_ is the leak conductance and *I* ^ext^ accounts for any external current onto the cable ^26^.

While the temporal part of the cable equation is emulated continuously on BSS-2, due to the physical implementation of the neurons, the space can not be modeled continuously. However, the spatial component of the cable equation can be discretized by using a second-order central difference on the second derivative with respect to space. This will lead to a multi-compartmental neuron model, which can be implemented on BSS-2, since single point neurons can be connected using conductances between them (compare Figure 1). We can extend the LIF neuron model to a multi-compartmental model, whose dynamics are given by:


$$\begin{array}{l} C_{\text{m},i}\frac{dU_{\text{m},i}(t)}{dt}=-g_{{}_{1,i}}\;\cdot \;(U_{\text{m},i}(t)-E_{1,i})\\ \;+\sum_{j}g_{ic,i↔j}\;\cdot \;(U_{\text{m},j}(t)-U_{\text{m},i}(t))+I_{i}^{\text{ext}}. \end{array}\;(2)$$

Here the second term on the right-hand side accounts for connections between neighboring compartments. In Equation 2, *C*_m,_ *_i_* describes the membrane capacitance of compartment *i*. The summation is performed over all neighboring compartments *j* of compartment *i*. *U*_m_, *_i_* is the membrane potential of compartment *i*, *E*_l, *i*_ is the leak potential, *g*_l, *i*_ the leak conductance, while *g*_ic, *i↔j*_ is the conductance connecting compartment *i* with a neighboring compartment *j*. $I_{i}^{\text{ext}}$ accounts for any external input current onto compartment *i* ^27^.

### Genetic algorithms

Genetic algorithms are used for search problems as well as optimization and belong to the class of evolutionary algorithms. As the name suggests, genetic algorithms are inspired by natural evolution ^28, 29^.

The first step of a genetic algorithm is to initialize random solutions to a problem. Each solution is called an individual. Individuals are constructed out of genes, which describe how the individuals solve the problem. Often genes are represented as arrays of numerical values. The collection of all individuals is referred to as population. In a sequential process, the population’s individuals are mutated, recombined and selected in order to create the next generation’s population. Usually, the selection is governed by the individual’s fitness, which is a score of how well the individual solves the problem. This process is repeated until an individual satisfyingly solves the problem or a predefined maximal number of generations is reached.

## Experiment

In this section, we explain the purpose and the setup of the experiment that will be used to investigate the genetic algorithm in the “results and discussion” section.

Our task is to tune a chain of compartments to match an observation. The observations are based on experiments that measured the decay of excitatory postsynaptic potentials (EPSPs) along a dendrite, see 30.

To replicate an attenuation experiment on BSS-2, first, we need to model the neuron’s morphology. Rather than modeling exact replicas of real neurons, we will only consider a linear chain of five compartments. This approach is sufficient for our purpose, as the focus of this paper is on the methodology of parametrizing the compartments.

**Figure 2.**
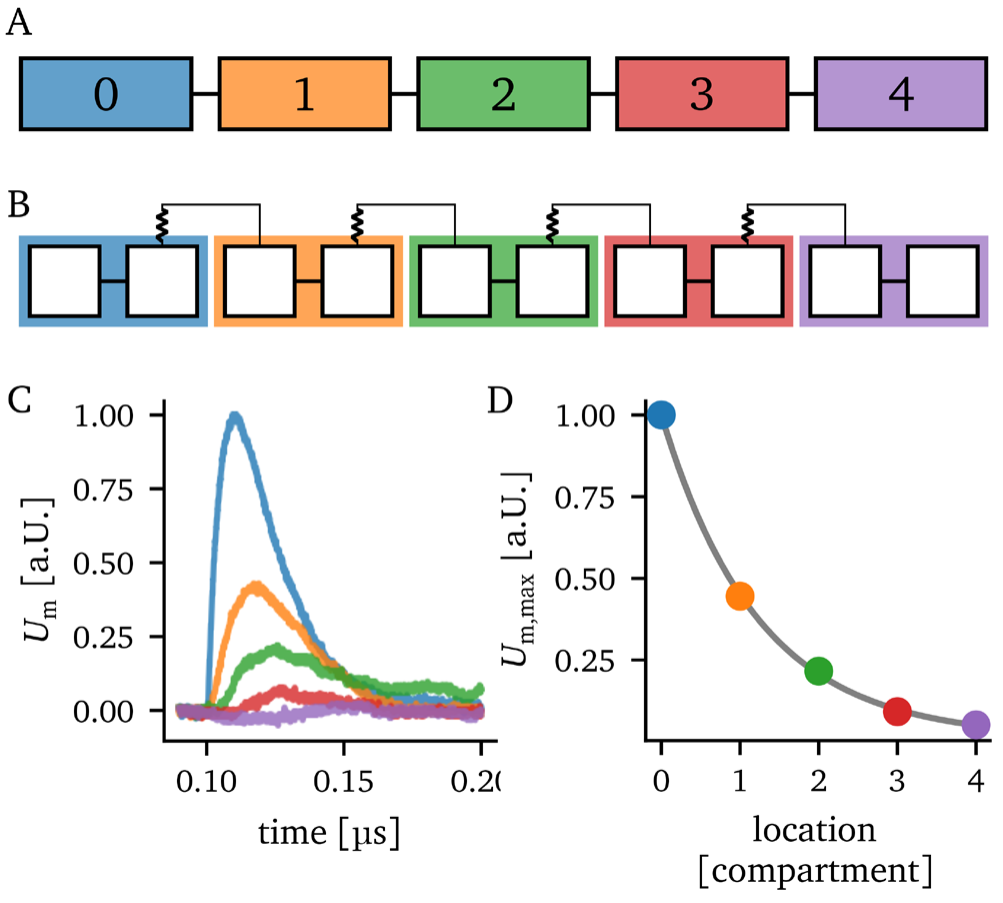
Experiment overview. **A**) Schematic of a linear compartment chain of length five. **B**) Possible realization of the compartment chain on BrainScaleS-2 using the same schematic as in Figure 1. **C**) Recordings of the excitatory postsynaptic potential (EPSP) in each compartment. The input is injected into compartment 0. Furthermore, the color of the EPSP traces indicate the compartment they belong to. **D**) Attenuation of the EPSP amplitudes along the chain of compartments. Additionally, an exponential of the form $U_{\text{m},\max}=a\cdot \exp(\text{−}x\text{/}\lambda_{\text{emp}})+c$ is fitted to the data.

A schematic of the considered compartment chain is given in Figure 2A alongside a possible implementation on BSS-2 in Figure 2B. By connecting the first compartment with a pre-synaptic partner, that elicits a spike, we can observe an EPSP. This EPSP travels along the chain of compartments, as shown in Figure 2C. The amplitude *U*_m,max_ of the EPSP within each compartment is shown in Figure 2D. Empirically we determined that the attenuation of the maximal EPSP height approximately obeys an exponential law with a length constant *λ*_emp_. A similar behavior was found in 30.

**Figure 3.**
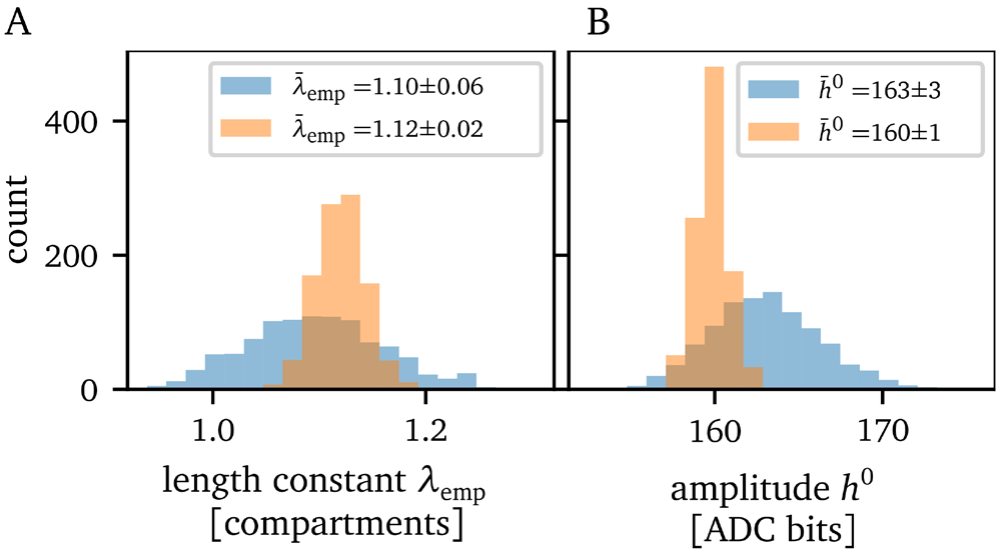
Trial-to-trial variations. **A**) Trial-to-trial variation of the length constant measurement and **B**) excitatory postsynaptic potential amplitude within the first compartment. The blue histograms show the single measurement scheme and the orange histograms show the results using the spike-triggered average. Both evaluation methods were executed 1000 times with the same parameterization of the chip.

Since we work with an analog substrate, trial-to-trial variations are inevitable. In Figure 3, the histograms illustrate the derivations between 1000 experiment runs using the same parameterization of the chip. Both the variation of the empirical length constant *λ*_emp_ and of the EPSP amplitude inside the first compartment are shown.

**Figure 4.**
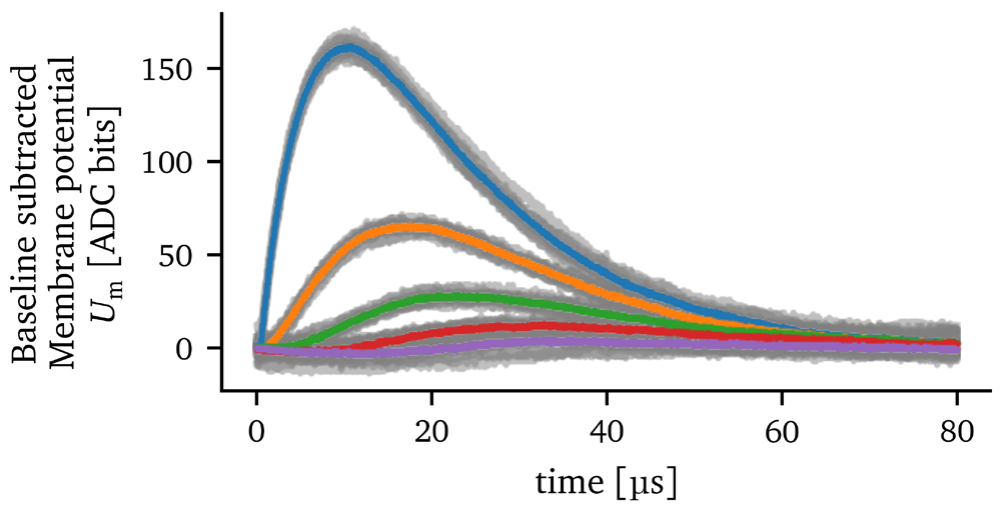
Spike-triggered average calculated from ten experiment runs. The color indicates the compartment where the excitatory postsynaptic potential was recorded (compare Figure 2). The traces of the single experiments are illustrated by the gray lines. For each run and compartment, the baseline is subtracted so that the resting potential is at zero.

To counteract the trial-to-trial variations, we use the spike-triggered average (STA) over several measurements ^31^. The STA describes the average membrane potential of a compartment in response to an input spike. Accordingly, we can modify the experiment by injecting *N*_spikes_ spikes with a time delay into the first compartment resulting in *N*_spikes_ EPSPs. The time delay between the spikes is set such that the membrane potential has enough time to return to its resting state. This way, multiple EPSPs can be recorded in one hardware run. For all results in this paper that use the STA, we will average the membrane potential over *N*_spikes_ = 10 input spikes, as this reduces the standard deviation of the trial-to-trial variations below the measurement resolution of the ADC. In Figure 4, the STA is calculated from ten experiment runs. The resulting traces are less noisy compared to the gray traces illustrating single experiment runs.

Ultimately, we can extract the amplitudes of the EPSPs from the STA and fit an exponential function to those amplitudes to obtain *λ*_emp_. This method decreases the standard deviation of the observables, which becomes apparent by the orange histograms in Figure 3. As a result, both observables exhibit a three times smaller standard deviation. Consequently, if not otherwise mentioned, all following results use the STA with *N*_spikes_ = 10. In Figure 3, we can see a small shift of approximately three bits towards smaller EPSP amplitudes. The STA helps to remove noise from the signal by smoothing it, which reduces the occurrence of slightly higher amplitudes caused by the noise of the membrane. Both recording methods agree within one standard deviation for the estimated length constant *λ*_emp_.

Recalling Equation 2, we can see that the dynamics of the membrane potential within each compartment depend on the membrane capacitance *C*_m, *i*_, the leak conductance *g*_l, *i*_ and the inter-compartment conductance *g*_ic, *i↔j*_. Therefore, we can change the attenuation behavior of the chain by changing these properties. Both the inter-compartment conductance and the leak conductance are adjustable using bias currents. Those bias currents are set by a digital-to-analog converter (DAC) with 10-bit resolution ^32^.

In order to get an overview of how these parameters influence the empirically found length constant *λ*_emp_, we sweep both the leak conductance and the inter-compartment conductance.

We extract the EPSP amplitude in each compartment for each parameter setting and fit an exponential function to the attenuation of the EPSP amplitudes (compare Figure 2D). This way, the empirical length constant *λ*_emp_ can be determined.

**Figure 5.**
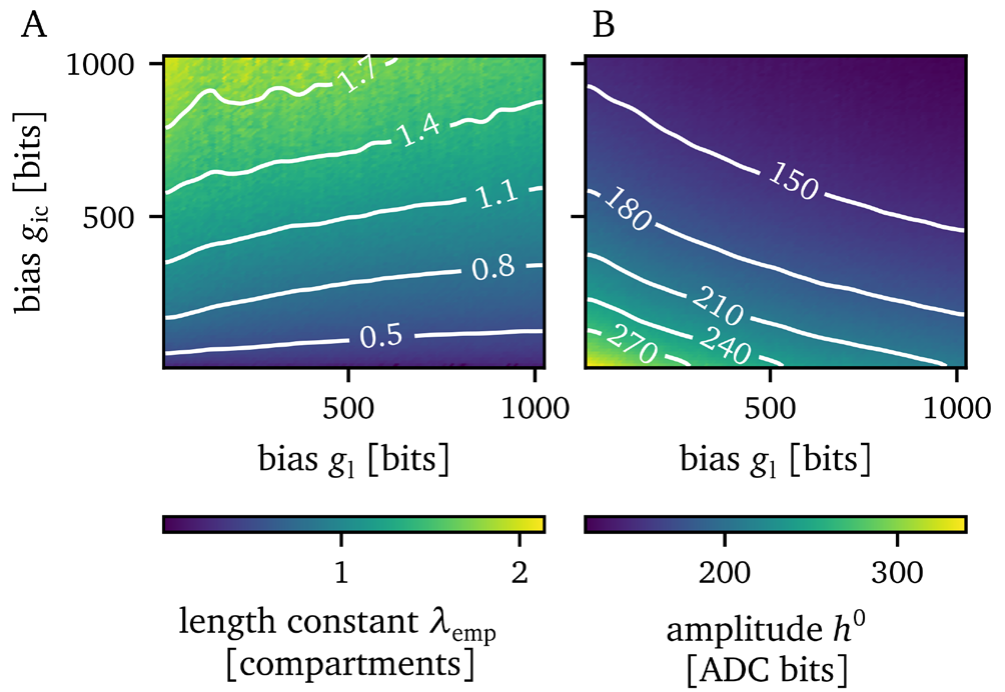
Grid search result over the whole parameter space. **A**) Empirically found length constant *λ*_emp_. **B**) Amplitude of the excitatory postsynaptic potential in the first compartment *h* ^0^.

Figure 5 shows that larger leak conductances will lead to smaller length constants, while larger inter-compartment conductances will increase the length constant. Additionally, the grid search indicates that there are multiple possible configurations of the pair of conductances resulting in the same length constant. Points in the solution space with the same length constant can be distinguished from one another by taking an additional observable into account, namely the amplitude of the resulting EPSPs. Since the strength of the synaptic event is constant, increasing the leak conductance will lead to a smaller EPSP. Likewise, increasing the inter-compartment conductance will lead to a smaller EPSP as well, as charge can more easily propagate to neighboring compartments. Consequently, increasing either the leak conductance or the inter-compartment conductance, will result in smaller amplitudes of the EPSP in the first compartment. This relationship is visualized in Figure 5B.

For our following investigations of the genetic algorithm, we pick one point in this parameter space, record it and extract a target observation for our algorithmic investigations. Then, the algorithm must find appropriate values for the inter-compartment and leak conductance that reproduce that observation. We pick the middle of the parameter space at a DAC value of 511 for both the leak conductance and the inter-compartment conductance to obtain our target observation, which will be the length constant with a value of ${\hat{\lambda }}_{\text{emp}}$ = (1.12 *±* 0.02) compartments.

This value was determined by taking the mean of 1000 measured EPSPs at the described fixed DAC values (compare Figure 3). For all other parameters the chip’s default calibration was used. The presented error describes the trial-to-trial variation calculated from the standard deviation of the different runs.

Furthermore, considering the amplitude of the EPSP, the problem can be extended to a multi-objective search problem.

In the next section, the used genetic algorithm will be introduced. 

## Methods

**Figure 6.**
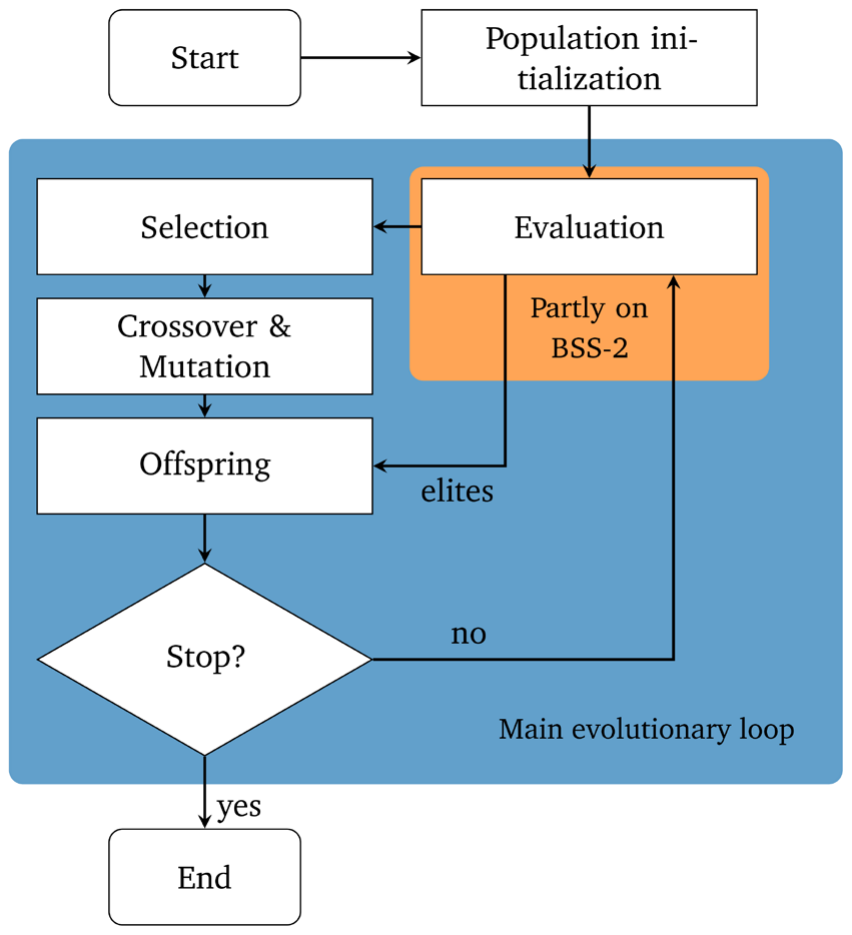
Flowchart of the used genetic algorithm with elitism. Only the evaluation step uses the neuromorphic hardware.

In this paper, the Python (RRID:SCR_008394) library DEAP ^1^ is used as backbone, which was developed for genetic algorithms with a high level of customizability ^33^. Figure 6 illustrates the procedural flow of our customized genetic algorithm.

The algorithm is based on DEAPs eaSimple ^34^ and extended to use an elitism mechanism ^35^. First, the algorithm randomly instantiates a set of *n*_individuals_ initial solutions. In our case, an individual will consist of two integer values, each describing a DAC setting. The first gene will describe the setting of the DAC responsible for the leak conductance and the second gene will describe the setting for the inter-compartment conductance. Because of that, the genes are bound to the value ranges of the respective DAC, which in our case is [0,1022] for both DACs.

Each individual’s fitness is determined in the evaluation step using the experiment described in the previous “experiment” section from which we obtain the corresponding length scale *λ*_emp_. By taking the difference between the measured length scale *λ*_emp_ and the desired target length scale ${\hat{\lambda }}_{\text{emp}}$ the fitness *f* can be calculated:


$$f=\left|\lambda_{\text{emp}}-{\hat{\lambda }}_{\text{emp}}\right|.\;(3)$$

As we are interested in individuals that produce observations that deviate as little from the target as possible we want to minimize this fitness *f*. The evaluation step is the only component of the genetic algorithm that executes instructions on the neuromorphic hardware.

Since we employ an elitism mechanism the *n*_elites_ fittest individuals are directly passed on to the next generation. The elitism mechanism ensures that the minimum fitness of each generation is not increasing.

Subsequently, the selection process begins, where we use tournament selection. Tournament selection randomly draws *k* individuals from the population, including the elites, and the individual with the best fitness score, *i.e.* the individual with the smallest fitness, will be selected. Since we use an elitism mechanism, we repeat the selection *n*_selection_ = *n*_individuals_ *−n*_elites_ times, where *n*_selection_ describes the number of selected individuals and *n*_elites_ is the number of elites.

After the selection process, the selected individuals are subject to evolutionary operations, like crossover and mutation. Two consecutive individuals are recombined with the probability *p*_cx_. The here used one-point crossover operator will recombine the genes (in our case, the single integer values of an individual) of two randomly picked individuals to create two offspring-individuals as depicted in Table 1.

**Table 1.** Example of one-point crossover applied to two parent individuals and the resulting offspring individuals.

gene	*g*_l_	*g*_ic_
parent 1	408	139
parent 2	364	975
offspring 1	364	139
offspring 2	408	975

Next, the mutation operator will apply point mutations on randomly chosen positions in the genome of the selected individuals with a fixed probability *p*_mut_. Here we use a custom mutation procedure: the gene’s new value is calculated by randomly adding or subtracting 2 *^x^* from the initial value, where *x* ∈ [0,9] is as well randomly chosen from a uniform distribution. If the new value falls outside the parameter boundaries, it will be rejected and a new value will be generated until it resides within the parameter boundaries.

Now, the procedure will start from the evaluation again if the stop criterion is not yet matched. In this implementation, we set a hard limit of 30 generation for the stop criterion. Finally, the fittest individual can be picked as a solution to the problem.

Several hyperparameters need to be set to run the algorithm. Unfortunately, there is no universal set of default hyperparameters that applies to every problem. Often the right hyperparameters are objective to trial-and-error methods and user experience. In Table 2, the used hyperparameters are summarized, which lead to sufficient results on our problem as can be seen in the next section. The hyperparameters are based on the findings in Keren *et al.* ^36^, Xia *et al.* ^37^.

**Table 2.** Hyperparameters used for the genetic algorithm to generate the presented results.

parameter	value	description
*n*_individuals_	50	Population size of each generation.
*n*_elites_	5	Number of elites directly passed to the next generation.
*n*_generations_	30	Number of generations to run.
Crossover	One-point	Cut genome at one point.
*p*_cx_	50%	Crossover probability.
Mutation	"Bit-flip"	Add/subtract 2 ^*x*^ to/from a gene.
*p*_mut_	10%	Mutation probability.
*p*_gen_	50%	Probability that a gene is mutated if individual is selected for mutation.
Selection	Tournament selection	Choose *k* individuals randomly and pick the fittest.
*k*	3	Tournament size.

## Results

In this section, we apply the just-described genetic algorithm to the experiment described in the “experiment” section and present the results.

**Figure 7.**
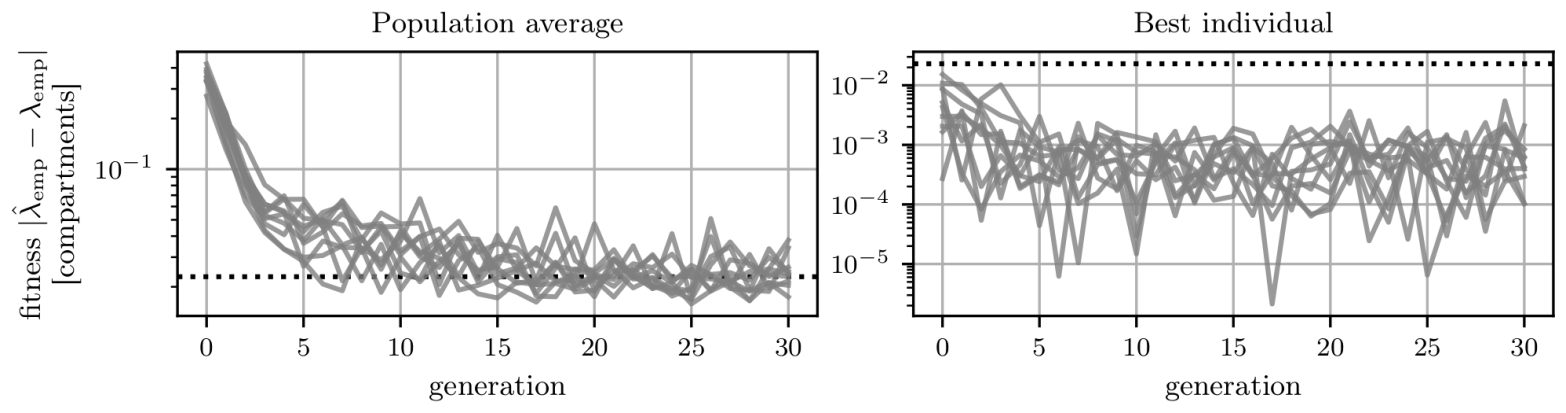
Fitness evolution of the genetic algorithm. The dashed black line indicates the error of the length scale measurement due to trial-to-trial variation. **A**) Average performance of the population in each generation. Each line indicates one of the ten runs. **B**) Performance of the best individual in each generation. Again each line represents one of the ten runs.

The algorithm was run ten times and the average fitness of the population and the fitness of the best individual in each generation are shown in Figure 7. The population’s average fitness is approaching the limit given by the standard deviation of the trial-to-trial variation of the searched-for target. However, there is always the chance that an evaluation of an individual leads to a length scale *λ*_emp_, which is closer than the trial-to-trial error to the target length scale ${\hat{\lambda }}_{\text{emp}}$ . This is the case for the best-performing individuals in Figure 7B. The fitness of the best individuals in all runs plateaus already after approximately five generations. This indicates that a solution is already found. Furthermore, trial-to-trial variations are the reason for fluctuations of the fittest individual within this plateau. This can be seen in Figure 7B, even though we employ an elitism mechanism, which on digital hardware would ensure that the best fitness does not increase ^2^.

**Figure 8.**
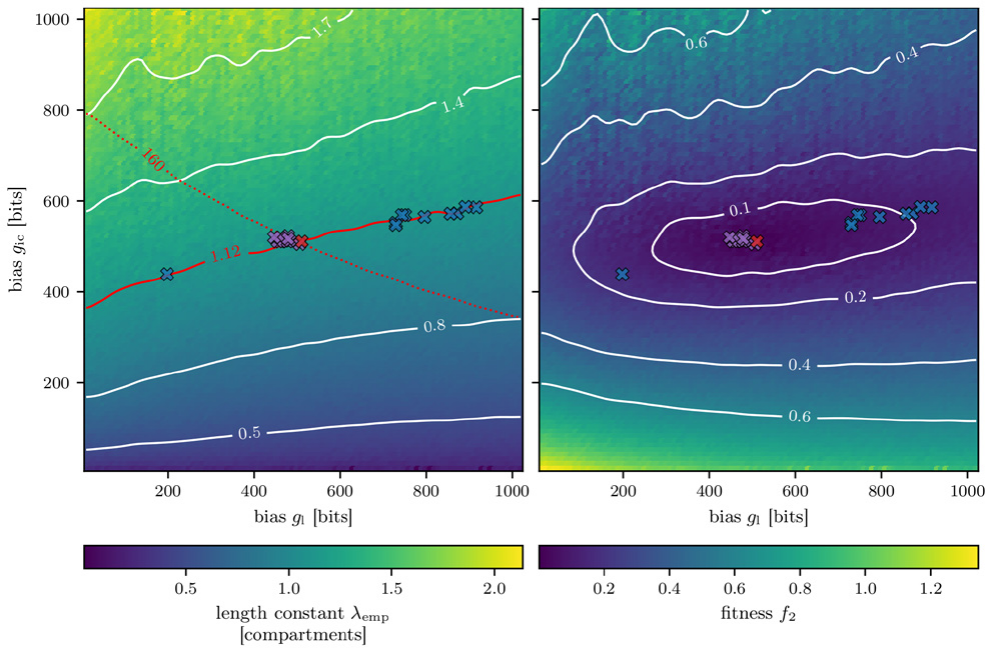
Solutions from ten runs using fitness function *f* (blue crosses) and *f*_2_ (purple crosses). **A**) The color of the background indicates the empirically found length constant *λ*_emp_ like in Figure 5A. The red line denotes the searched for target length scale of ${\hat{\lambda }}_{\text{emp}}$ = 1.12 compartments, and the red cross the parameterization which resulted in the searched for target length scale. The red dotted line illustrates the corresponding target amplitude of the excitatory postsynaptic potential in the first compartment ${\hat{h}}^{0}$ of 160 bits. The blue crosses show the final solutions of the genetic algorithm with fitness function *f* from Equation 3, while the purple crosses exhibit the solutions of the genetic algorithm using the fitness function *f*_2_ from Equation 4. **B**) Here the background shows the fitness landscape, which was calculated using the grid search result from Figure 5 and the fitness function from Equation 4.

Next, in Figure 8, the resulting parameters of the single algorithm runs are visualized. The red cross illustrates the parameters which were used to create the target length scale ${\hat{\lambda }}_{\text{emp}}$ . The blue crosses represent the solutions of the genetic algorithm. All ten solutions are in the vicinity of the red contour line, which marks the length scale of 1.12 compartments. Since the problem was to find the length constant, all provided solutions are valid.

By considering the amplitude *h* ^0^ of the EPSP in the first compartment of the chain for the fitness evaluation we can change the fitness landscape.

This will further reduce the space for valid solutions since a solution must now match *both* the searched-for length scale *λ*_emp_ and the EPSP amplitude of the first compartment *h* ^0^. An example of an adapted fitness function, considering the length constant *λ*_emp_ and the EPSP amplitude *h* ^0^, can be expressed as:


$$f_{2}=\sqrt{{\left(\frac{{\hat{\lambda }}_{\text{emp}}-\lambda_{\text{emp}}}{{\hat{\lambda }}_{\text{emp}}}\right)}^{2}+{\left(\frac{{\hat{h}}^{0}-h^{0}}{{\hat{h}}^{0}}\right)}^{2}}.\;(4)$$

Here ${\hat{h}}^{0}$ is the target amplitude of the EPSP in the first compartment, which is (160 *±* 1) bits. This value is the average measured amplitude for the EPSP at a DAC setting of 511 for both the leak and inter-compartment conductance (compare Figure 3).

The fitness landscape of the fitness function *f* from Equation 3 would be minimal along the red contour line of Figure 8A and would increase on both sides of it. Using the fitness function *f*_2_, the fitness landscape changes according to the shape depicted in Figure 8B. Now solutions with minimal fitness can only be found around the target marked by the red cross.

Rerunning the genetic algorithm ten times with the adapted fitness function *f*_2_ results in the solutions marked by the purple crosses in Figure 8.

Now all solutions are much closer to the red cross marking our target setting.

## Conclusions

In this paper, we have demonstrated that genetic algorithms can be utilized to parameterize analog neurons on BSS-2 such that high-level observations can be replicated. We parameterized two neuron parameters such that the desired attenuation behavior of an EPSP along a compartment chain could be replicated.

First, the trial-to-trial variations of experimental observations due to the analog nature of the used hardware were quantified and used as an estimate for the error. Employing STA reduced the trial-to-trial variation of different experiment runs. Then, through a grid search, the solution space was visualized.

The solutions produced by the genetic algorithm replicated observations within the error boundaries defined by the observed trial-to-trial variations. Additionally, the genetic algorithm could coupe with an extension to the problem, making it a multi-objective search. The optimizer was able to simultaneously minimize the distance to a target length constant ${\hat{\lambda }}_{\text{emp}}$ and a target EPSP amplitude within the first compartment ${\hat{h}}^{0}$ . Overall genetic algorithms could profit twofold from accelerated analog neuromorphic hardware, first through the speed gain and second through its intrinsic parallelization capabilities as genetic algorithms are predestined for running in parallel. Neurons on BSS-2 possess membrane time constants a thousand times smaller than their biological archetypes. Therefore, each evaluation of an individual profits from that speed-up, especially for long experiment run times.

Genetic algorithms can be parallelized since the evaluation of an individual is independent of other individuals. Therefore, the whole population can be evaluated at once. On BSS-2, there are 512 neuron circuits so multiple experiments can be run in parallel. However, there is fixed-pattern variation between neuron circuits. The same bias current applied to different neuron circuits can therefore lead to slightly different observations. Especially, the effectiveness of the crossover operation would suffer from that. Nevertheless, this problem can be circumvented by working on model parameters, which in turn requires a parameter transformation using chip-specific calibration data.

For example, in our experiment, the leak conductance *g*_l_ and the inter-compartment conductance *g*_ic_ could have functioned as universal parameters. Finally, this would allow a translation from one neuron circuit to another and therefore allow parallelization.

## Ethics and consent

Ethical approval and consent were not required.

## Data Availability

The underlying data can be accessed via heiDATA: https://doi.org/10.11588/data/U2U1IB/KUM85K ^38^. The archived folder contains all experiment results. Data are available under the terms of the Creative Commons Attribution 4.0 International license (CC-BY 4.0). Following the instructions in the corresponding repository, https://github.com/electronicvisions/model-paper-mc-genetic, the underlying data also contains all information to reproduce the experiment results and generate the plots presented in this publication. The BSS-2 system can be accessed *via* EBRAINS.

## Software availability

**Source code available from:** https://github.com/ electronicvisions/model-paper-mc-genetic.

**Archived source code at the time of publication:** https://doi.org/10.5281/zenodo.8109123 ^39^.

**License:** LGPL-2.1 license.
