# Supplementary material for: Parametrizing analog multi-compartment neurons with genetic algorithms
Source: Open Res Eur. 2024 Nov 14;3:144. Originally published 2023 Sep 8. [Version 2] doi: 10.12688/openreseurope.15775.2 (PMC11635192; doi:10.12688/openreseurope.15775.2)
Supplement: Peer review history

# Reviewer report: Reviewer response for version 2

**DOI:** 10.21956/openreseurope.20368.r46276

**Published:** 2025-01-17

**Authors:** Wenhao Song

**Affiliations:** 1 University of Southern California, Los Angeles, California, USA

I appreciate the authors' efforts in performing extended experiments with nine parameters. The extended experiments in Figures 9 and 10 provide more solid evidence of the effectiveness of the genetic algorithm. Unsurprisingly, there will be a discrepancy between the targets and observations in Figure 10 due to the hardware device-to-device variations and the authors discussed it. The concern is whether this inaccuracy would become more and more severe as the parameter space scales up or if there is a way to control it. The second concern is about the efficiency of the genetic algorithm. Grid search is extremely inefficient, and beating grid search is not strong enough evidence to prove the practiceness of the proposed algorithm. Adding a little more discussion and comparison on the efficiency of the algorithm would be great.

Is the study design appropriate and does the work have academic merit?

Partly

Is the work clearly and accurately presented and does it cite the current literature?

Yes

If applicable, is the statistical analysis and its interpretation appropriate?

Yes

Are all the source data underlying the results available to ensure full reproducibility?

Yes

Are the conclusions drawn adequately supported by the results?

Partly

Are sufficient details of methods and analysis provided to allow replication by others?

Yes

Reviewer Expertise:

neuromorphic computing, in-memory computing, device and circuit modeling, memristors.

I confirm that I have read this submission and believe that I have an appropriate level of expertise to confirm that it is of an acceptable scientific standard.

# Reviewer report: Reviewer response for version 2

**DOI:** 10.21956/openreseurope.20368.r47743

**Published:** 2025-01-09

**Authors:** Arianna Rubino

**Affiliations:** 1 University of Zurich and ETH Zurich, Zurich, Switzerland

This work proposes to use genetic algorithms to parametrize configurable components on the BrainScaleS-2 hardware.

 The BrainScaleS-2 chip enables the creation of complex multi-compartment neurons by interconnecting simple individual neurons. In this paper, the authors exploit this feature to design a chain of compartments to replicate observed EPSP attenuation phenomena. Genetic algorithms are employed to optimize neuron model parameters, specifically leak and inter-compartment conductances, to accurately replicate the EPSP attenuation experiment.

 The effectiveness of genetic algorithms for parameterizing analog neurons on the BrainScaleS-2 chip is demonstrated across problems with both low- and high-dimensional parameter spaces.

 The manuscript is clear, detailed, and well-structured. It provides sufficient background to understand the study, and the methods and results sections are comprehensive and easy to follow. Additionally, the authors present results obtained from a neuromorphic hardware and share the necessary data and source code to facilitate experiment replication.

 I have only one suggestion and curiosity. The authors justify their choice of genetic algorithms by claiming (and reporting previous related work) that they are well-suited for parameterizing multi-compartment neuron models and outperform other optimization methods. However, it would strengthen the study to include a comparison of the genetic algorithm’s performance with other optimization techniques for this specific experiment. Furthermore, it would be interesting to know more details on the time required for the genetic algorithm to complete the parameterization.

Is the study design appropriate and does the work have academic merit?

Yes

Is the work clearly and accurately presented and does it cite the current literature?

Yes

If applicable, is the statistical analysis and its interpretation appropriate?

Yes

Are all the source data underlying the results available to ensure full reproducibility?

Yes

Are the conclusions drawn adequately supported by the results?

Yes

Are sufficient details of methods and analysis provided to allow replication by others?

Yes

Reviewer Expertise:

Neuromorphic mixed-signal circuit design, Spiking Neural Networks

I confirm that I have read this submission and believe that I have an appropriate level of expertise to confirm that it is of an acceptable scientific standard.

# Reviewer report: Reviewer response for version 2

**DOI:** 10.21956/openreseurope.20368.r47157

**Published:** 2024-12-20

**Authors:** Erika Covi, Andrea Giuntoli, Nayan Vengallur

**Affiliations:** 1 University of Groningen, Groningen, The Netherlands

This work uses genetic algorithms to find the model parameters for multi-compartment neuron models. The model is then physically implemented and demonstrated on the BrainScaleS-2 hardware.

 The manuscript is clear, well written, logically organised and well structured. The work is well motivated and the results are fully satisfactory.

 The hardware implementation is very solid and well described. The motivation for using genetic algorithms for this type of problem is convincing.

 There are only two details missing that should be included in the manuscript for better clarity of the impact of this work:

 1) it is not very clear how an element of the population is encoded precisely, meaning how the set of impedances is uniquely codified into a string that defines a member of the population. 

 2) as a consequence of (1), the dimensional space explored by the genetic algorithm (i.e., the number of possible different population elements) is not easy to extract from the paper. A brief and more explicit discussion on this would be relevant, especially in the context of quantifying how big their "high-dimensional" case is, and the efficiency in convergence time of the GA compared to the estimated time needed to find the same solution via a grid search.

Is the study design appropriate and does the work have academic merit?

Yes

Is the work clearly and accurately presented and does it cite the current literature?

Yes

If applicable, is the statistical analysis and its interpretation appropriate?

Yes

Are all the source data underlying the results available to ensure full reproducibility?

Yes

Are the conclusions drawn adequately supported by the results?

Yes

Are sufficient details of methods and analysis provided to allow replication by others?

Partly

Reviewer Expertise:

Analogue circuit design

We confirm that we have read this submission and believe that we have an appropriate level of expertise to confirm that it is of an acceptable scientific standard.

# Reviewer report: Reviewer response for version 2

**DOI:** 10.21956/openreseurope.20368.r47745

**Published:** 2024-12-11

**Authors:** Tanguy Damart

**Affiliations:** 1 Ecole Polytechnique Federale de Lausanne, Lausanne, Vaud, Switzerland

Constraining the parameters of a neuron model using an optimisation algorithm is a well-established method. In the present paper, the authors demonstrate that this method can be applied to a multi-compartment dendrite model implemented on a neuromorphic platform (BrainScaleS-2).

 The authors created a five-compartment model of a dendrite, each including a leak and inter-compartment conductance. Performing a grid search, they showed how the amplitude and spatial attenuation of an EPSP vary as a function of these two parameters and discussed in detail the trial-to-trial variability inherent to the use of a neuromorphic platform.

 In a second experiment, the authors used an evolutionary strategy to show that starting from a random set of solutions, an optimiser can converge toward a solution reproducing the EPSP properties observed for one parameter set obtained from the grid-search experiment.

 Overall the work is interesting, and I recognize the effort that went into it. Constraining the parameters of neuron models is a computer-intensive task and making use of neuromorphic computing could be fruitful in that regard.

 Despite that, I have reservations that need to be addressed before recommending this work for indexing.

 The present manuscript has been proposed to Open Research Europe as a Research Article, as such, we expect an in-depth discussion of the scientific relevance of the work. However, the manuscript does not discuss the scientific motivation for the optimisation of dendritic parameters on neuromorphic hardware. More specifically:

1. The motivation for the use of BrainScaleS-2 for parameter optimisation seems to be the speed gain mentioned by the author in the introduction. However, in the rest of the manuscript, no discussion describing the effective speed-up brought by the use of such a platform versus traditional hardware is present. Without the characterisation of the advantages brought by neuromorphic hardware, the question of whether the use of such hardware is relevant for parameter optimisation stays unanswered.
2. The present authors made the choice of using an elitist genetic algorithm as an optimizer. However, the motivation for the choice of such an algorithm is missing. As illustrated in Fig 7, genetic algorithms present an inherent lack of convergence once a low-fitness subspace of the parameter landscape has been reached and, as such, are not necessarily a good fit for well-behaved problems. Other methods, such as Newtonian methods or covariant matrix adaptation, have to be benchmarked to evaluate their competitiveness on the present problem.

Is the study design appropriate and does the work have academic merit?

Partly

Is the work clearly and accurately presented and does it cite the current literature?

Partly

If applicable, is the statistical analysis and its interpretation appropriate?

Not applicable

Are all the source data underlying the results available to ensure full reproducibility?

Yes

Are the conclusions drawn adequately supported by the results?

Yes

Are sufficient details of methods and analysis provided to allow replication by others?

Yes

Reviewer Expertise:

spiking neural networks, detailed neuron modeling, multi-objective optimisation methods

I confirm that I have read this submission and believe that I have an appropriate level of expertise to confirm that it is of an acceptable scientific standard, however I have significant reservations, as outlined above.

# Reviewer report: Reviewer response for version 1

**DOI:** 10.21956/openreseurope.17040.r38498

**Published:** 2024-04-19

**Authors:** Charles P. Rizzo

**Affiliations:** 1 The University of Tennessee Knoxville, Knoxville, Tennessee, USA

The authors demonstrate how genetic algorithms can be employed to parameterize multi-compartmental neurons on analog neuromorphic hardware. They provide an overview of how the BrainScaleS-2 neuromorphic chip works as it is relevant to their experiments, their neuron model, and how genetic algorithms work.

 They perform an experiment, using spike-triggered averaging (STA) to counteract trial-to-trial variations, in which they derive the empirical length constant and the excitatory postsynaptic potential amplitude (EPSP) within the first compartment of their evaluated 5-compartment chain. They then employ a genetic algorithm to optimize the values for both inter-compartment and leak conductance to reproduce their observed length constant measurement.

 Overall, the experiment is interesting, well-formed, and a great use of genetic algorithms. Their methods are sound, and sufficient details to reproduce the experiment are provided. Their chosen hyperparameters and experiment length are appropriately justified. All of their figures are drawn well and help the reader further understand the experiment. Their results are also displayed well, match expectations, and are thoroughly discussed in the text. Additionally, they take a step further and present a multi-objective fitness function that reduces the valid solutions space, and they highlight better results when using said fitness function.

 The only small point of confusion I had was in parsing Figure 7B, and trying to work out how the best individual's fitness for each generation in each run was not consistently decreasing or constant (given that the authors employed an elitism mechanism), but had I read the rest of the paragraph preceding Figure 7B. first, I'd have noticed that the authors address this. 

 I have no reservations in strongly recommending this work for indexing, and I applaud the authors on a job well done.

Is the study design appropriate and does the work have academic merit?

Yes

Is the work clearly and accurately presented and does it cite the current literature?

Yes

If applicable, is the statistical analysis and its interpretation appropriate?

Not applicable

Are all the source data underlying the results available to ensure full reproducibility?

Yes

Are the conclusions drawn adequately supported by the results?

Yes

Are sufficient details of methods and analysis provided to allow replication by others?

Yes

Reviewer Expertise:

neuromorphic computing, spiking neural networks, event-based cameras, genetic algorithms

I confirm that I have read this submission and believe that I have an appropriate level of expertise to confirm that it is of an acceptable scientific standard.

# Response

**Published:** 2024-11-15

**Authors:** Raphael Stock

**Affiliations:** Physics, Universitat Heidelberg, Heidelberg, Baden-Württemberg, Germany

We thank Charles P.Rizzo for his review and positive feedback.

 In order to avoid the confusion regarding figure 7B, we added an additional sentence to the caption.

 This should make the figure more self-contained.

# Reviewer report: Reviewer response for version 1

**DOI:** 10.21956/openreseurope.17040.r38499

**Published:** 2024-04-19

**Authors:** Wenhao Song

**Affiliations:** 1 University of Southern California, Los Angeles, California, USA

The authors Raphael *et al.* introduced an example that using genetic algorithms to find the optimal hardware parameters on the BrainScaleS-2 neuromorphic hardware platform. More specifically, they used multiple neurons in BrainScaleS-2 to build a multi-compartmental neuron, and tried to determine the best parameters that give the desired length constant and the EPSP amplitude. There are only two parameters in the search space, the leak conductance g_l and the inter-compartment conductance g_ic.

 Overall, this manuscript is well written with adequate or even abundant details explained. The figures are of proper form and drawn with high quality. And there are many highlights in this work: BrainScaleS-2 is a novel platform with lots of potentials, and the multi-compartment neuron models can capture more important details compared with point neuron models.

 The relative weakness is mainly about the method used for this optimization: the genetic algorithms. Genetic algorithm is a classic algorithm that performs well when we don’t have any prior knowledge. As long as it can be proven suitable for this task, this manuscript can still be of high quality.

1. The efficiency of the genetic algorithm. “we chose genetic algorithms since they outperformed conjugate-gradient descent and stochastic search as demonstrated by Vanier and Bower7 in a task to constrain a multi-compartmental neuron model.” However, this reference was in 1999 and we believe both methods have been developed further and new methods including machine learning have been developed as well. A more recent or direct comparisons on the efficiency of possible algorithms are desired to maintain this assertion’s validity.
2. The necessity of using genetic algorithms, or the scale of the problem solved. With only 2 parameters (g_l ad g_ic) to be optimized and at most 2 objectives, the parameter space can be easily thoroughly search as shown in Fig 5. This cannot show the advantages of genetic algorithms, which is more suitable for more parameters. Moreover, the objectives shown in Fig 5 or 7B are quite simple with only one minimal, which may just be solved by conjugate gradient without much problem. In other words, genetic algorithm may be an overkill for a simple parameter search problem proposed in the manuscript. Therefore, a larger experiment with more parameters to be optimized would be desired to show the necessity and advantage of the genetic algorithm used. For similar reasons, the claimed “speed gain” lacks the data support from comparison.

Is the study design appropriate and does the work have academic merit?

Partly

Is the work clearly and accurately presented and does it cite the current literature?

Yes

If applicable, is the statistical analysis and its interpretation appropriate?

Yes

Are all the source data underlying the results available to ensure full reproducibility?

Yes

Are the conclusions drawn adequately supported by the results?

Partly

Are sufficient details of methods and analysis provided to allow replication by others?

Yes

Reviewer Expertise:

neuromorphic computing, in-memory computing, device and circuit modeling, memristors.

I confirm that I have read this submission and believe that I have an appropriate level of expertise to confirm that it is of an acceptable scientific standard, however I have significant reservations, as outlined above.

# Response

**Published:** 2024-11-15

**Authors:** Raphael Stock

**Affiliations:** Physics, Universitat Heidelberg, Heidelberg, Baden-Württemberg, Germany

We thank Wenhao Song for the thorough revision and the helpful feedback.

 In the following we address the comments by Wenhao Song and state what improvements we implemented in the presented manuscript. 1. We added two more recent publications which also employed genetic algorithms and compared them to different optimization methods.

 Furthermore, the explicit mention of "conjugate-gradient descent" was misleading.

 We rephrased the paragraph to make more clear that we chose genetic algorithms because of their competitive performance compared with other algorithms and since they are frequently used to optimize the parameters of multi-compartmental neuron models.

 Furthermore, we chose genetic algorithms since they were previously used in combination with neuromorphic hardware.

 This is mentioned in the next paragraph of the manuscript. 2. We agree that for a two-dimensional parameter space genetic algorithms do not show their advantages.

 Therefore, we extended the experiment to optimize nine parameters in parallel.

 In case of a grid search with ten points per parameter, a nine-dimensional problem would require 10^9 measurements.

 The results we added to the manuscript show that the genetic algorithm is able to find suitable solutions in less then 3000 (60 generations, 50 individuals) measurements.

 Since we performed additional experiments for the current revision, we updated our code and performed all experiments again.

 Due to the analog nature of the BrainScaleS-2 system, the new software state and a different calibration, the exact values differ slightly form the previous results.

 The main results and conclusions are the same.

 We updated the figures in the manuscript and created a new version of the data set (which is shared via heiDATA and referenced in the paper).
